# The prevalence of depression and its associated factors among patients with diabetes mellitus attending a tertiary clinic in Gaborone, Botswana

**DOI:** 10.4102/sajpsychiatry.v28i0.1647

**Published:** 2022-02-28

**Authors:** Thato Moshomo, Yordanka Pina Rivera, Judith Boshe, Godfrey M. Rwegerera

**Affiliations:** 1 Department of Internal Medicine, Faculty of Medicine, University of Botswana, Gaborone, Botswana; 2 Department of Medicine, Sidilega Private Hospital, Gaborone, Botswana; 3 Department of Psychiatry, Kilimanjaro Christian Medical Centre, Moshi, Tanzania; 4 Department of Medicine, Princess Marina Hospital, Gaborone, Botswana

**Keywords:** depression, diabetes mellitus, prevalence, glycaemic control, Botswana

## Abstract

**Background:**

Depression is one of the commonest co-existing medical conditions among patients with diabetes mellitus (DM). A bidirectional relationship between depression and DM exists, complicating glycaemic control leading to an increase in diabetic complications. There is a dearth of information regarding the prevalence of depression and associated factors among patients with DM in Botswana.

**Aim:**

This study aimed to determine the prevalence of depression and associated factors among patients with DM. The study also assessed the association between depression and glycaemic control.

**Setting:**

A tertiary diabetic referral clinic in Gaborone, Botswana.

**Method:**

A sample of 260 randomly selected patients with DM was recruited in this cross-sectional study. Socio-demographic and clinical characteristics of the patients were collected using a case report form. Depression was evaluated using the Patient Health Questionnaire (PHQ)-9 scale. Multivariate regression analysis was used to determine factors significantly associated with depression.

**Results:**

The mean age (standard deviation [s.d.]) of study participants was 58.4 (11.8) years, and the majority, 160/260 (61.5%), were females. The prevalence of depression was 30.4% and significantly associated with female sex (adjusted odds ratio [AOR] = 5.529, *p*-value = 0.004), three or more diabetes-related hospitalisations (AOR = 3.886, *p*-value = 0.049) and inversely associated with systolic blood pressure (SBP) ≥ 140 mmHg (AOR = 0.11, *p*-value = 0.001).

**Conclusion:**

Depression is a common problem among patients with DM in our setting. Routine screening of depression in diabetic patients to enable early detection and treatment is recommended.

## Introduction

Diabetes mellitus (DM) is an important public health concern with a high prevalence of co-existing medical conditions.^1^ Recent data from the International Diabetes Federation (IDF) show a prevalence of diabetes of 415 million in 2015 and estimated to reach 642 million by 2040.^2^ Global estimates further show that over 70% of people with DM are likely to live in developing countries, particularly sub-Saharan Africa (SSA).^3,4^ According to the American Psychiatric Association *Diagnostic and Statistical Manual of Mental Disorders fifth edition* (*DSM-5*), depression is defined as a mood disorder that comprises several physical, cognitive and emotional symptoms that impair an individual’s daily functioning.^5^ There is good evidence that psychosocial matters are important to improving self-blood glucose monitoring practices, diet and medication adherence.^6,7^ Poor self-care (e.g. lack of adherence to lifestyle recommendations and glucose monitoring) is driven by depression and contributes to poor glycaemic control and diabetic complications over time.^8,9^ A recent meta-analysis of 248 studies by Khaledi et al. revealed a significant association between depression and diabetes with a worldwide prevalence of comorbid major depressive disorder at 28%.^10^ Older meta-analysis and global studies have also shown rates of major depressive disorder at 12% and depressive symptoms at 15%–35% among individuals with DM.^8,9,11,12^ Despite the fact that poor self-care practices among patients with DM contribute to poor glycaemic control over time, depression has also been shown to contribute to poor glycaemic control through activation of stress pathways with increased levels of cortisol, adrenaline and noradrenaline resulting in poor glycaemic control.^13^ The bidirectional association between DM and depression involves complex mechanisms, whose understanding provides room for the better treatment approaches and improves the outcomes for these two pathologies.^13,14^

Recent studies showed that there are not any common genetic factors to account for the positive association between depression and type 1 or 2 DM.^15^ However, different environmental factors (epigenetic factors) may activate common pathways that promote type 2 diabetes and depression in the end. One important factor is a low socioeconomic status that increases the odds for type 2 diabetes^13,16^ but also appears to be a cause for depression.^13,17^ The other common relationship between DM and depression is poor sleep, lack of physical exercises and poor adherence to eating habits or self-care activities.^14,18^ A key candidate for a common pathway could be the activation and disturbance of the stress system. Chronic stress activates the hypothalamus–pituitary–adrenal axis (HPA-axis) and the sympathetic nervous system (SNS), increasing the production of cortisol in the adrenal cortex and the production of adrenaline and noradrenaline in the adrenal medulla.^13,19^ Chronic hypercortisolemia and prolonged SNS activation promote insulin resistance and visceral obesity and lead to metabolic syndrome and type 2 DM.^20^ In contrast, chronic stress has behavioural consequences: noradrenaline, cortisol and other hormones activate the fear system determining anxiety and anorexia; the same mediators cause suppression of the reward system, which produces depression and cravings for food, other substances or stress.^20^ Moreover, chronic stress induces immune dysfunction directly or through the HPA axis or SNS, increasing the production of inflammatory cytokines such as interleukin-6 (IL-6), interleukin-1 (IL-1) and tumour necrosis alpha (TNF-alpha), which in high amounts induce insulin resistance and ultimately type 2 DM.^21,22^

Previous studies in different parts of the world have explored factors associated with depression among patients with DM in outpatient settings and identified several demographic, behavioural, and clinical factors that predispose diabetic patients to depression.^23,24^ These include female gender, older age, lower levels of education,^25^ being single or unmarried^26^ and high body mass index (BMI).^23^ Other behavioural and clinical factors associated with increased risk of depression among patients with DM include social isolation, monthly household income, starting antidiabetic drugs at a young age^27^ and presence of long-term DM complication such as nephropathy.^28^

Evidence from multiple systematic reviews and randomised controlled studies involving large sample size of patients has shown that recognising depression and providing psychological plus pharmacological interventions results in clinically significant improvements in both depression symptoms and glycaemic control.^29^ There is limited data on the prevalence of comorbid depression and diabetes in SSA. A meta-analysis of nine studies with 2944 participants in Ethiopia revealed a prevalence of depression among DM patients of 39.73%.^30^ Studies with fewer participants included cross-sectional studies in Nigeria,^31^ Tanzania,^32^ Malawi^33^ and a Kenyan prospective study^34^ with the prevalence of 30%, 30%, 18% and 20.9%, respectively. In contrast, two studies conducted in South Africa yielded higher prevalence rates of 46% and 46.6%, respectively.^35,36^ The literature search did not reveal any publication on the association between diabetes and depression in Botswana. The study aimed to establish the prevalence of depression and its associated factors among diabetic patients attending a tertiary clinic in Gaborone, Botswana. It also compared the psychometric properties of the two depression screening tools: Patient Health Questionnaire (PHQ)-9 and Beck’s Depression Inventory (BDI)-II, among patients with DM in our setting.

## Materials and methods

### Study design and setting

A cross-sectional study was conducted at a tertiary diabetic clinic between 10 June 2019 and 30 August 2019. The clinic is situated in Gaborone, the capital city of Botswana and offers several services including consultation, dietician services, eye examinations and pharmacy. It acts as a referral clinic for patients with diabetic complications and poor glycaemic control from local clinics within Gaborone and neighbouring towns in Southern, Central and Eastern Botswana. The choice of this clinic makes it appropriate for this study given that it serves as a main tertiary clinic for diabetes in Botswana, and findings are likely to represent the population of DM that is difficult to treat in Botswana.

### Participants

We recruited patients who were 21 years of age or older attending the diabetic clinic during the study period and having had the diagnosis of DM for at least 3 months and who consented to participate in the study. Patients with underlying severe mental illness such as schizophrenia or inability to comprehend the explained content of the study that made them unable to consent were excluded.

### Sample size calculation and sampling

A minimum sample size of 246 participants was required as ascertained using a formula for cross-sectional study.^37^ The prevalence (P) of depression among diabetic patients of 20% from a study in Jordan^38^ that used the PHQ-9 was utilised to obtain our sample size. It should be observed that this was a Middle Eastern study; it would have been ideal to use prevalence from SSA studies; however, at the time of protocol development, there were no similar published studies in SSA using PHQ-9. We considered using the prevalence of 41% by the study of Udedi et al. in Malawi;^39^ however, this prevalence was obtained using Structured Clinical Interview for *DSM-IV* (SCID) for depression tool and not PHQ-9. We added 5% from the obtained sample size anticipating missing variables because of recall or missing results reaching 258, which was rounded to the final sample size of 260 participants. The order of recruitment was randomised on each day. Five pieces of papers of equal sizes and shapes were numbered 1–5 for patients to pick and identify the first patient to be recruited on each recruitment day. On each clinic day, about 60–70 patients attend for consultations. Our intention was to recruit at least seven patients per day; hence every eighth eligible patient was systematically recruited.

### Ethical considerations

The study protocol was approved by the Ministry of Health and Wellness Research Unit and all relevant Institutional Review Boards in Gaborone, Botswana (IRB). All recruited participants signed written informed consent forms. Consent included assessing participants’ information from their outpatient charts and medical electronic system. It was made clear for each participant that there was no remuneration, and there was no physical harm to the participants. To ensure confidentiality, the interview was conducted in a secured consultation room with only the researcher and participant present. The recruited participants were assigned unique reference numbers for the study. There was also a pre-emptive plan to inform the attending physicians and urgently refer patients with moderately severe and severe depression (PHQ-9 ≥ 15) or active suicidal ideations to a psychiatric facility.

### Procedures

One bilingual Motswana nurse translated the socio-demographic and clinical section of the case report form into the Setswana. This was followed by an evaluation of the translated tool by the first author. Two bilingual Batswana nurses then back translated the Setswana version into English independently and then met with the first author to arrive at the consensus translations.

Patient Health Questionnaire-9 has been previously used in Botswana in Setswana language^40^; hence the Setswana version was adopted for this study. We did not have permission to translate the BDI-II inventory tool; hence the English version was used throughout.

Data were collected by the first author with the assistance of a trained research assistant who was a paramedic with experience of working as an assistant in clinical research. The research assistant was trained on the details of the research prior to data collection initiation. Training entailed interpretation of socio-demographic, clinical and depression variables. Informed written consent was obtained from each patient before the interview and study procedures. The study was introduced to all the potential participants whilst awaiting consultations on each day of recruitment. This was followed by approaching each participant separately for eligibility and consent. Efforts were made to recruit participants whilst awaiting their turn for physician’s consultation; however, in few occasions, participants were recruited after physician’s consultation. Data were captured through face-to-face interviews using a case report form to obtain socio-demographic and clinical characteristics of the study participants. Socio-demographic variables collected included age, gender, level of education, marital status, household size and main source of household income, income per month and employment status. Clinical variables obtained included the following information: type of diabetes, duration of diabetes, type of antidiabetic drugs in use, number of diabetes-related hospitalisations, presence of documented complications in patients’ chart (retinopathy, nephropathy, cardiomyopathy, peripheral neuropathy, peripheral vascular disease, erectile dysfunction), number of documented complications, history of hypertension (yes/no), documented human immunodeficiency virus (HIV) status within past 1 year (positive/negative/unknown).

### Study definitions

Participants were categorised to have type 1 DM if they were diagnosed to have DM before the age of 30 years, and insulin was their first antidiabetic medication^41^ and was documented to have type 1 DM in their charts. Diabetes-related hospitalisation was defined according to ICD 10 coded E10–E14 and referred to admission because of coma with/without ketoacidosis, hyperosmolar coma and hypoglycaemic coma, renal, ophthalmic, neurologic and peripheral circulatory complications.^42^ Hypertension was defined using Joint National Committee (JNC) 8 as systolic blood pressure (SBP) of ≥ 140 mm Hg and/or diastolic blood pressure (DBP) of ≥ 90 mm Hg^43^ in at least two prior visits or by current use of antihypertensive drugs. Depression was a binary measure similar to previous studies with PHQ-9 cut-offs of ≥ 10 indicating the presence of depression whilst < 10 was regarded as the absence of a diagnosis of depression.^44^ For BDI-II, cut-off of ≥ 20 referred to depression, whereas < 20 indicated no depression.^45^ Patient Health Questionnaire-9 was used to assess for the presence and severity of depression for all the recruited participants.

### Depression assessment tools

There is no validated tool to screen for depression in Botswana, and a formal diagnosis of depression requires a validated interview, but this can be time consuming.^46^ As a result, quick and inexpensive methods are available to screen people in primary and secondary care settings.^47^ Many short questionnaires have been used to screen for depression in the general population, but only a few have been evaluated adequately in people with DM.^48^

Some of the most well-validated depression screening questionnaires sharing psychosomatic properties for people with DM include the Beck Depression Inventory,^45^ the Centre for Epidemiologic Studies Depression Scale,^49^ the PHQ^50^ and the Hospital Anxiety and Depression Scale (HADS).^51^

Based on the fact that the PHQ-9 had not been validated in Botswana at the time of conducting the study, Beck’s Depression Inventory-II inventory was also administered for the last 100 patients. The two tools were used for convergent analysis, allowing exploration of psychometric properties of the tools.

Patient Health Questionnaire-9 tool is free for use and does not require permission for copyright use. This tool has been validated internationally and in some sub-Saharan African countries such as Malawi, South Africa, Kenya, Zimbabwe and Mozambique.^39,52,53,54,55,56^ The authors paid for the cost of 100 BDI II English version forms purchased from Pearson.

### Clinical evaluation

Physical examination of patients included measuring the weight and height of each patient. Weight and height were routinely measured during registration using a stadiometer when the participant was on light clothing without shoes. Body mass index was calculated as a ratio of weight (kg) divided by height^2^ (m^2^) and categorised as underweight (< 18.5), normal weight (18.5–24.9), overweight (25–29.9) and obese (≥ 30). Body mass index categorisation was performed according to the World Health Organization (WHO).^57^ Blood pressure measurements were taken for each patient during registration. A second blood pressure measurement was carried out by the research assistant; the mean of the two blood pressures was used during analysis. Categorisation of blood pressure was performed according to JNC 8 guidelines for optimal blood pressure treatment goals in patients with diabetes as ≥ 90 and ≥ 140 mmHg for poorly controlled DBP and SBP, respectively. In contrast, < 90 and < 140 mmHg referred to good control of DBP and SBP, respectively.^43^

### Glycaemic control

Glycosylated haemoglobin (HbA1c) is a measure of average glucose control over the preceding 3 months. Treatment goals for participants with DM were categorised as follows: desirable (< 7%), suboptimal (7% – 9%) and poor (≥ 9%).^58^ Glycosylated haemoglobin (HbA1c) (performed within 3 months of index visit) was obtained from the computer system (Integrated Patients Management System [IPMS]) or patients’ files. If the patient had no recent result, the HbA1c was ordered on the day of interview (the test was performed free of charge).

### Data analysis

Data collected were entered and cleaned with Statistical Package of Social Sciences (SPSS) version 20 followed by analysis using the R version 3.6.1 and Stata 14.0. Frequencies were used to describe the demographic and clinical characteristics of the respondents. To determine the appropriate summary statistics, continuous distributions were checked for normality using the Shapiro–Wilk’s test. If the normality assumption was met, the mean and standard deviation (s.d.) were used to summarise the data, whereas the median and interquartile ranges (IQR) were used for variables that were not distributed normally. The Cronbach’s alpha statistic was computed to determine the internal consistency of PHQ-9 score and BDI II in terms of measuring depression, whereas convergence validity to obtain a correlation of two tools was computed by William’s test. Internal consistency equal or above 0.7 was considered acceptable.^59^ Odds ratios (ORs) were used to test associations between a binary dependent variable (depression) using both PHQ-9 score and independent variables (socio-demographic, clinical characteristics and glycaemic control). Bivariate and multivariate logistic regression analyses were used for evaluating the factors associated with depression. Only variables that were significant at 10% level of significance in the bivariate logistic regression were used in the multivariate regression model.

The stepwise model selection technique was used to pick only variables that were relevant to the model. A *p*-value of less than 0.05 was considered to be statistically significant.

## Results

A total of 260 participants were randomly selected and included in the final analysis. The average age of study participants was 58.4 years (s.d. = 11.8), with the majority being females, 160/260 (61.5%). More than half of the participants, 159/260 (59.2%) in this study were earning the lowest of incomes (less than Botswana Pula [BWP] 3000 per month/approximately $277.05). The rest of the socio-demographic characteristics is shown in Table 1.

**TABLE 1:** Socio-demographic characteristics of study participants.

Demographics	Frequency (*n*)	%
**Age in years** (Mean ± s.d.)	58.4 ± 11.81	-
21–40	20	7.7
41–60	120	46.2
> 60	120	46.2
**Gender**		
Male	100	38.5
Female	160	61.5
**Level of education**		
No formal school	42	16.2
Primary school or less	115	44.2
Secondary school completed	73	28.1
College/university/post-graduate degree	30	11.5
**Marital status**		
Never married	100	38.5
Currently married	119	45.8
Separated	2	0.8
Divorced	2	0.8
Widowed	25	9.6
Cohabiting	12	4.6
**Source of household income**		
Self	158	60.8
Spouse	39	15.0
Others	63	24.2
**Income per month**		
Less than BWP3000	154	59.2
BWP3000–BWP8000	86	33.1
BWP8000–BWP15 000	19	7.3
More than BWP15 000	1	0.4

s.d., standard deviation; BWP, Botswana Pula.

The majority of study participants were documented to have type 2 DM, 251/260 (96.5%). In contrast, over two-thirds of participants had been diabetic for 5 years or more, 175/260 (67.3%). Over half of the participants were either overweight or obese, 145/260 (55.8%) whereas 74.6%, 77.3% and 72.0% of study participants were HIV-negative, hypertensive and with at least one documented diabetic complication, respectively. Peripheral neuropathy and diabetic eye complications were the most commonly documented complications accounting for 60.8% and 57.7%, respectively (Table 2).

**TABLE 2:** Clinical characteristics of study participants.

Variables	Frequency (*n*)	%
**Type of DM**		
Type 1	9	3.5
Type 2	251	96.5
**Duration of DM in years (median [IQR])**	6	4–10
< 5	85	32.7
≥ 5	175	67.3
**Modality of treatment for diabetes**		
Non-insulin	155	59.6
Insulin injection	105	40.0
**History of hypertension**		
Yes	201	77.3
No	59	22.7
**Number of documented DM complications**		
None	57	22.0
1–2	172	60.4
≥ 3	30	11.6
**Number of DM-related hospitalisations**		
< 3	80	86.0
≥ 3	13	14.0
**HIV status within past 1 year**		
Positive	30	11.5
Negative	194	74.6
Unknown	36	138
**BMI**		
Underweight (< 18.5)	7	2.7
Normal weight (18.5–24.99)	108	41.5
Overweight (25–29.99)	88	33.8
Obese (≥ 30)	57	21.9
**SBP in mmHg**		
Good control (SBP < 140)	114	43.8
Poor control (SBP ≥ 140)	146	56.2
**DBP in mmHg**		
Good control (DBP < 90)	223	85.8
Poor control (DBP ≥ 90)	37	14.2

DM, diabetes mellitus; IQR, interquartile range; BMI, body mass index; SBP, systolic blood pressure; DBP, diastolic blood pressure.

The mean glycaemic control (HbA1c) of the study participants was 7.4% (s.d. = 6.5% – 8.8%) with 162/260 (62.3%) of participants having suboptimal or poor glycaemic control (Table 3).

**TABLE 3:** Glycaemic control of study participants.

Glycaemic control variable	Frequency (*n*)	%
**HbA1c %** (mean ± s.d.)	7.4	6.5–8.8
Optimal (< 7)	98	37.7
Sub-optimal (7–9)	106	40.8
Poor (>9)	56	21.5

s.d., standard deviation.

The prevalence of depression as measured by PHQ-9 was found to be 30.4% (79 participants). Beck’s Depression Inventory-II that was administered for the last 100 participants revealed a prevalence of depression of 27.0% (Table 4). There were no participants with active suicidal thoughts; however, 12 of the 79 participants with depression using PHQ-9 met criteria for further expert evaluation (moderately severe with score 15–19 and severe with a score of 20–27) with or without active suicidal thoughts; hence they were appropriately referred to the psychiatry and psychology departments.

**TABLE 4:** Prevalence of depression among study participants.

Depression status	PHQ-9; Frequency (*n*)	%	BDI II; Frequency (*n*)	%
No depression	181	69.6	73	73.0
Depression	79	30.4	27	27.0

PHQ, Patient Health Questionnaire; BDI, Beck’s Depression Inventory.

Bivariate logistic regression analysis was used to determine the association between depression and socio-demographic characteristics. There was no significant difference in depression status across different age groups. Female participants were more likely to be depressed compared with their male counterparts (OR = 2.558, 95% CI = 1.4–4.6; *p*-value = 0.002). Depression was more likely to be found among participants with lower education status, with the likelihood of being depressed decreasing in more literate participants. Participants with a tertiary education qualification were significantly associated with less depression (OR = 0.294, 95% CI = 0.94–0.92, *p*-value = 0.036). Participants who were never married were more likely to be depressed compared with those who were married (77% vs. 65.5%); however, the difference was not statistically significant (*p*-value = 0.065). In contrast, analysis of the level of income showed participants who earned the lowest income (less than BWP3000 per month/approximately $277.05) were more likely to be depressed as compared with counterparts who earned more monthly income (BWP3000–BWP8000/approximately $277.05–$738.79 and more than BWP8000 (approximately $738.79); (35.7% vs. 24.4% vs. 15.8%, respectively). The association between level of income and depression status was, however, not statistically significant (Table 5).

**TABLE 5:** The association between depression (using Patient Health Questionnaire-9) and socio-demographic characteristics.

Demographics	Depression (*n*)	%	No depression (*n*)	%	Unadjusted OR (95% CI)	*p*
**Age in years**						
21–40	6	30	14	70	1	-
41–60	37	30.8	83	69.2	1.04 (0.371–2.919)	0.940
> 60	36	30	84	70	1 (0.356–2.809)	1
**Gender**						
Male	19	19.0	81	81.0	1	-
Female	60	37.5	100	62.5	2.558 (1.413–4.630)	0.002
**Level of education**						
No formal	17	40.5	25	59.5	1	-
Primary school or less	37	32.2	78	67.8	0.698 (0.336–1.447)	0.334
Secondary school	20	27.4	53	72.6	0.555 (0.249–1.238)	0.150
Tertiary education	5	16.7	25	83.3	0.294 (0.240–0.920)	0.036
**Marital status**						
Never married	23	23	77	77	1	-
Currently married	41	34.5	78	65.5	1.760 (0.966–3.206)	0.065
Others	15	36.6	26	63.4	1.931 (0.878–4.247)	0.102
**Source of income**						
Self	48	30.4	110	69.6	1	-
Others	31	30.4	71	6.6	1.001 (0.582–1.719)	0.998
**Income per month**						
Less than BWP3000	55	35.7	99	64.3	1	-
BWP3000–BWP8000	21	24.4	65	75.6	0.582 (0.322–1.051)	0.073
More than BWP8000	3	15.8	17	84.2	0.318 (0.089–1.132)	0.077

OR, odds ratio; CI, confidence interval; BWP, Botswana Pula.

Participants with three or more diabetes-related hospitalisations were significantly more likely to be depressed compared with those with fewer diabetes-related hospitalisations (OR = 3.50 (95% CI = 1.052–11.645, *p*-value = 0.041). In contrast, participants who recorded high SBP of ≥ 140 mmHg were less likely to have depression as compared with those who recorded normal SBP (OR = 0.502 [95% CI = 0.294–0.857, *p*-value = 0.012]). The rest of the studied clinical characteristics including duration of DM, a modality of treatment, number of documented diabetic complications, history of hypertension, HIV status and BMI were not associated with depression (table not shown).

Participants with no documented peripheral neuropathy were less likely to be depressed as compared with those with documented peripheral neuropathy (OR = 0.487, 95% CI = 0.275–0.864, *p*-value = 0.014). The rest of the diabetic complications was not associated with depression status (table not shown).

There was a tendency of patients being more depressed as HbA1c increased. Patients with poor glycaemic control (HbA1c) >9% were statistically more likely to be depressed compared with those with good glycaemic control in bivariate analysis (OR = 3.380, 95% CI = 1.647–6.938, *p-*value = 0.001). Glycaemic control using either fasting blood glucose or random blood glucose was not associated with depression status (*p*-value = 0.947; table not shown).

The variables that fitted the final stepwise approach are shown in Table 6. Female gender (adjusted odds ratio [AOR] = 5.529, 95% CI = 1.749–17.485, *p*-value = 0.004) and three or more diabetes-related hospitalisations (AOR = 3.886, 95% CI = 1.008–14.972, *p*-value = 0.049) were significantly associated with having depression. Systolic blood pressure of ≥ 140 mmHg remained statistically significantly associated with less proportions of depression (AOR = 0.11, 95% CI = 0.03–0.39, *p*-value = 0.001; Table 6).

**TABLE 6:** Multivariate logistic regression showing the association between depression status and socio-demographic and clinical characteristics/complications.

Characteristics	Adjusted OR (95% CI)	*p*
**Gender**		
Male	1	-
Female	5.529 (1.749–17.485)	0.004
**No. of diabetes-related hospitalisations**		
< 3	1	-
≥ 3	3.886 (1.008–14.972)	0.049
**SBP in mmHg**		
Controlled (< 140)	1-	-
Poor control (≥ 140)	0.175 (0.059–0.521)	0.002

OR, odds ratio; CI, confidence interval; SBP, systolic blood pressure.

**FIGURE 1:**
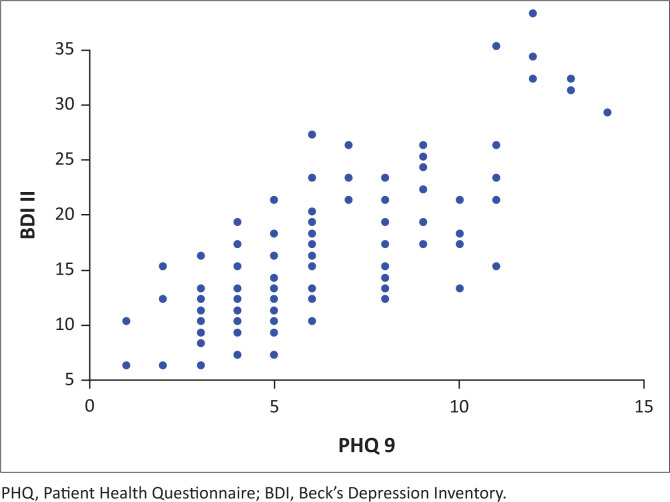
Scatter plot of Beck’s Depression Inventory II against Patient Health Questionnaire-9.

The Cronbach’s alpha of 0.896 was obtained when comparing PHQ-9 and BDI II (Table not shown) indicating that the two tools have good internal consistency. There was a strong positive correlation (*r*) between PHQ-9 and BDI II as revealed in the scatter plot in Figure 1. Reinforcing the significant positive linear correlation was a correlation (*r*) = 0.79 (*p*-value = 0.001; table not shown), indicating that the two tools have comparable convergence.

## Discussion

The prevalence of depression in our study in Botswana that used PHQ-9 cut-off ≥ 10 was found to be 30.4% indicating that almost a third of screened patients were depressed. This is almost similar to the findings of a recent meta-analysis of 248 studies showing a worldwide prevalence of 28%.^10^ It is, however, slightly lower than findings of a meta-analysis of nine studies in Ethiopia where the majority of the studies used PHQ, with a prevalence of 39.73%^30^; the difference observed may have resulted from the difference in definition of depression as some studies in the meta-analysis used PHQ cut-off of ≥ 5. Comparable findings were observed in some sub-Saharan countries such as Nigeria^31^ and Tanzania^32^ both at 30%. The prevalence of depression in our study differs from some sub-Saharan countries: South Africa (46%, 46.6%),^35,36^ Kenya (20.9%),^34^ Malawi (18%)^33^ and some Non-African countries: Jordan (19.7%)^38^ and Australia (20.8%).^60^ The wide variation in prevalence of depression between this study and others may be explained by several factors including differences in socio-demographic factors, study designs, depression assessment tools and sample size differences. Differences in culture and religion may also contribute to variations in prevalence of depression between countries.^61,62^ Societal norms such as focusing on physical symptoms of depression only with less emphasis on emotional aspects may also explain some differences.^62^ Evidence also shows that illness perceptions and thus their expressions may differ across cultures.^62^ In some societies and especially for older people, being depressed is considered as ‘normal’.^62^

Female gender was found to be significantly associated with depression both in bivariate and multivariate logistic regression. Similar findings have been observed across the majority of other studies.^10,12,14^ Mental health problems including depression are more commonly observed in females than males in various communities and social contexts.^25^ This may be because of the burden brought by their multiple roles and responsibilities, lack of employment, sex discrimination and associated factors such as gender-based violence that contributes to women’s poor mental health.^63^

Bivariate analysis revealed that higher education levels decreased the odds of being depressed; this is similar to several other studies.^28,30^ Education is thought to improve or expose an individual towards a better understanding of disease mechanisms and complications, leading to improved adherence towards disease treatment for better health outcomes. However, after a stepwise multivariate analysis, this effect was not observed; a possible explanation for this being that the sample size for the study might have been small to detect a difference in a multivariate model.

Age was not associated with depression in this study. This finding is similar to what was observed in other studies performed in Nigeria^64^ and South Australasia.^25^ However, it contrasts with a cross-sectional study among hospitalised patients with Type 2 DM at a tertiary hospital in Saudi Arabia, which found an increased risk of depression with older age.^28^ The difference may be because of different patient population characteristics.

Previous studies consistently showed that being single or unmarried is associated with an increased likelihood of having depression.^26,64^ Single participants appeared to be more likely to be depressed as compared with those married in this study; however, the association was not statistically significant. The lack of significant association between marital status and depression found in this study is similar to findings from other studies.^29^

A higher level of income in this study appeared to be associated with less likelihood of having depression; this is similar to previous studies^25,29^; however, this was not statistically significant probably because of sample size effect. It should also be observed that other studies that used smaller sample sizes than this study in Nigeria^64^ and Morocco^65^ also found no association between level of income and depression.

Bivariate and multivariate analyses showed a significant association between being depressed and an increased number of DM-related hospitalisations. This was consistent with one large study in Australia.^66^ This association may imply hospitalisations for acute complications such as diabetic ketoacidosis (DKA) or hyperosmolar hyperglycaemic state (HHS) precipitated by poor self-care in diabetic patients. Hospitalisation may also be related to chronic complications arising from poor glycaemic control observed in these depressed patients.

Bivariate and multivariate analysis revealed an inverse relationship between SBP control and depression. The results of this study are similar to what was reported in a study in Norway, which showed that depressed patients not on antidepressants were likely to have lower blood pressure (both DBP and SBP) than their controls.^67^ As the association of blood pressure and depression was only significant for SBP, but not DBP in our study, future studies must be conducted on this aspect to enhance our understanding.

Although the presence of diabetic complications has been shown to be associated with depression^12^ most of the studied DM complications in this study were not associated with depression. The most likely explanation for this is that the real extent of diabetic complications might have been underestimated because the study only counted documented complications; no active physical examinations, investigation or screening were performed on that respect. Other studied clinical characteristics included duration of diabetes, modality of diabetes treatment and history of hypertension and BMI. All these variables were not associated with depression contradicting previous studies that showed duration of diabetes, being on insulin and BMI to be associated with depression.^23^ In contrast, lack of association between duration of diabetes and insulin use in this study is consistent with a study carried out in Pakistan.^24^

Given the high prevalence of HIV in Botswana and the fact that a previous study indicated over a quarter of studied participants living with HIV to be depressed,^68^ it was important to assess the significance of HIV status as a contributor to depression. Human immunodeficiency virus infection was not associated with depression in this study. A possible explanation for this finding is that patients who attended at the tertiary diabetic clinic were well-controlled immunologically and virologically and hence less likely to be depressed compared with those 18–49 years recruited in a population-based study in districts for the previous study of depression among patients living with HIV in Botswana. Likewise, this study recruited fewer HIV-infected patients and hence may be less powered to detect any association. This lack of HIV association was also observed in the Kenyan study.^34^

Our bivariate analysis findings confirm and extend findings of previous studies by showing that depression is significantly associated with poor glycaemic control. This is consistent with numerous studies.^11^ Glycosylated haemoglobin (HbA1c) of more than 9% was associated with depression; although this was not significant in multivariate analysis, it is of clinical significance and should be explored further in future local studies with larger sample sizes. Depressed mood can affect glycaemic control through at least two plausible mechanisms: either through changes in self-care or through counter-regulatory hormones or physiological disturbances. Several studies have shown an association between levels of depression and diabetes self-care. Ceichanowski et al.^69^ found that depression was associated with poorer diet, medication adherence and physical activity levels. Similarly, Lustman et al.^8^ found that depression was associated with poorer blood glucose self-monitoring. Depression-associated non-adherence was also observed in a meta-analysis of 47 studies.^70^ This, therefore, suggests that depressed mood could reduce appropriate self-care behaviours, which would negatively impact glycaemic control.

Reliability analysis comparing PHQ-9 and BDI II revealed good internal consistency, and there was a strong linear positive correlation between the two depression screening tools. The PHQ-9 categorised a slightly greater proportion of patients with major depression than the BDI-II. These results indicate that despite both tools not being validated in this setting they can both be used to screen for depression among patients with DM in Botswana.

## Limitations

The cross-sectional nature of the study does not provide a causal. Furthermore, the study may be prone to recall and reporting bias because some data were collected based on past and self-reported information. This could affect the nature of the relationships between depressive symptoms on the one hand and self-reported complications or medications on the other. Moreover, as the data were gathered from a single facility, findings may not be generalisable to all DM patients in Botswana, more especially those in the private sector or those in rural areas with no comprehensive diabetic care. In contrast, patients referred or being managed at this tertiary facility are those who cannot be managed at primary care clinics because of poorly controlled DM and that may have an impact on the experience of depression. This study excluded patients aged 18–20 years who also fall within the consenting age in Botswana; this is because most of the patients in this age group continue to follow up for their diabetic care at the adolescent clinic in the main referral hospital in Gaborone, Botswana. Despite the stated limitation, this is the first study to explore depression among DM patients in Botswana, and it offers important knowledge to build on future-related studies in Botswana and similar settings in SSA.

## Conclusion

The results of this study have shown that depression is a fairly common and under-recognised comorbidity among patients with DM in Botswana. It is an important piece of information to diabetes specialists, physicians, psychiatrists and general practitioners attending patients with DM to emphasise the need for routine screening of depression among patients with DM to enable early detection and treatment. Patients at risk for depression, including females, poor glycaemic control, those with three or more diabetes-related hospitalisations and lower SBP, should be prioritised. There is a need for future prospective and interventional studies across multiple facilities in Botswana to enhance more understanding of depression and its effect on morbidity and mortality of DM patients. Patient Health Questionnaire-9 depression screening tool was shown to have good internal consistency and strong positive linear correlation when compared with BDI II supporting its use in future studies.
